# Atypical Cogan's Syndrome

**DOI:** 10.1155/2013/476527

**Published:** 2013-04-04

**Authors:** João Queirós, Sofia Maia, Mariana Seca, António Friande, Maria Araújo, Angelina Meireles

**Affiliations:** Departmento de Oftalmologia, Hospital de Santo António, Centro Hospitalar do Porto, Largo Professor Abel Salazar, 4099-001 Porto, Portugal

## Abstract

*Background*. Cogan's syndrome is a rare clinical entity whose etiopathology is still unknown, and the treatment strategies are not clearly defined. *Case*. A 23-year-old male presented with symptoms of headache, peripheral facial palsy, persistent right hearing loss and bilateral papillitis. Workup excluded all infectious, granulomatous, neoplastic, and immune causes. The diagnosis of atypical Cogan's syndrome was established, and the patient was treated with systemic corticosteroids and later on with cyclophosphamide and methotrexate. There were improvement of visual symptoms and stabilisation of left hearing. *Conclusion*. Cogan's syndrome is a very rare disease with no specific biological tests for the diagnosis. The diagnostic exams are mostly important to exclude other etiologies. The atypical ocular and audiovestibular manifestations make the diagnosis difficult, delaying the institution of appropriate therapy which may result in profound bilateral deafness.

## 1. Introduction

Cogan's syndrome (CS) is a very rare disorder of unknown etiology, most commonly observed in young Caucasian adults of either gender. It was described in 1945 by Cogan as an association of nonsyphilitic interstitial keratitis (IK) and Ménière-like audiovestibular symptoms [1]. Later, in 1980, Haynes and colleagues suggested that the condition should include other ocular and audiovestibular symptoms and proposed diagnostic criteria for typical and atypical CS [2].

In the typical form, the ocular involvement is characterized by IK, sometimes associated with conjunctivitis, subconjunctival haemorrhage, or iritis. On examination, an irregular, granular, perilimbic corneal infiltrate is observed. In most cases, both eyes are affected during the disease course. In the atypical form, many ocular lesions have been described, either isolated or associated with IK, including episcleritis/scleritis, retinitis, choroiditis, optic neuritis, papillitis, and central retinal artery occlusion, among others.

The typical ear involvement resembles a picture of Ménière disease with a rapid onset of vertigo, nausea, vomiting, or tinnitus, accompanied with progressive hearing loss, usually evolving to deafness in 3 months. The hearing loss is often bilateral from the onset of the disease, but in some patients it may be unilateral, becoming bilateral later on [3]. In the atypical form, the audiovestibular symptoms are not characteristic of a Ménière-like disease.

We report a case of an atypical Cogan's syndrome, the workup of the diagnosis, and treatment results.

## 2. Case Report

A 23-year-old male was referred to the emergency room with a 3-month history of peripheral facial palsy, hearing loss, bilateral optic disc edema, and headaches refractory to painkillers in the last few weeks. He had been treated with oral prednisone for the facial palsy, with improvement of symptoms, but when the drug was being tapered, he developed hearing loss and decreased visual acuity.

At presentation, he had a mild left facial palsy with lagophthalmos, a severe impairment of right hearing, and several papulovesicular lesions on the face and back. 

**Figure 1:**
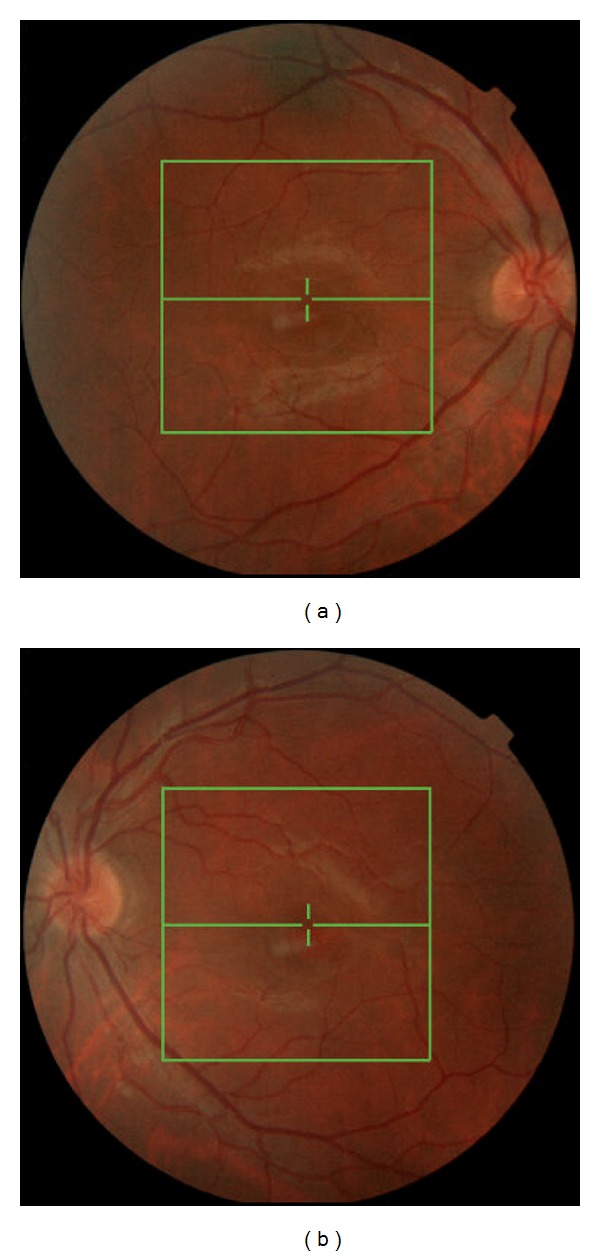
Retinography showing mild hyperemia of both optic discs.

On ophthalmic examination, the best corrected visual acuity (BCVA) was 20/32 in the right eye and 20/20 in the left eye. The anterior segment examination was normal, and intraocular pressure was 13 mmHg in both eyes. The fundus examination revealed mild hyperemia of both optic discs, with blurred borders, and no signs of vitreous or retinal inflammation (Figure 1). No other neurologic signs or symptoms were found.

From the baseline blood investigation performed, only the erythrocyte sedimentation rate (ESR) and the C-reactive protein (CRP) were abnormal (ESR of 27 mm/h; CRP of 33,25 mg/L). From the infectious and immunological panel, there was only a positive Weil-Felix reaction.

The cerebral magnetic resonance imaging (MRI) revealed a cranial multineuritis involving the right II cranial nerve and III, V, VII, and VIII nerves. All the microbiologic and serologic tests from the spinal tap were negative and the opening pressure was normal.

**Figure 2:**
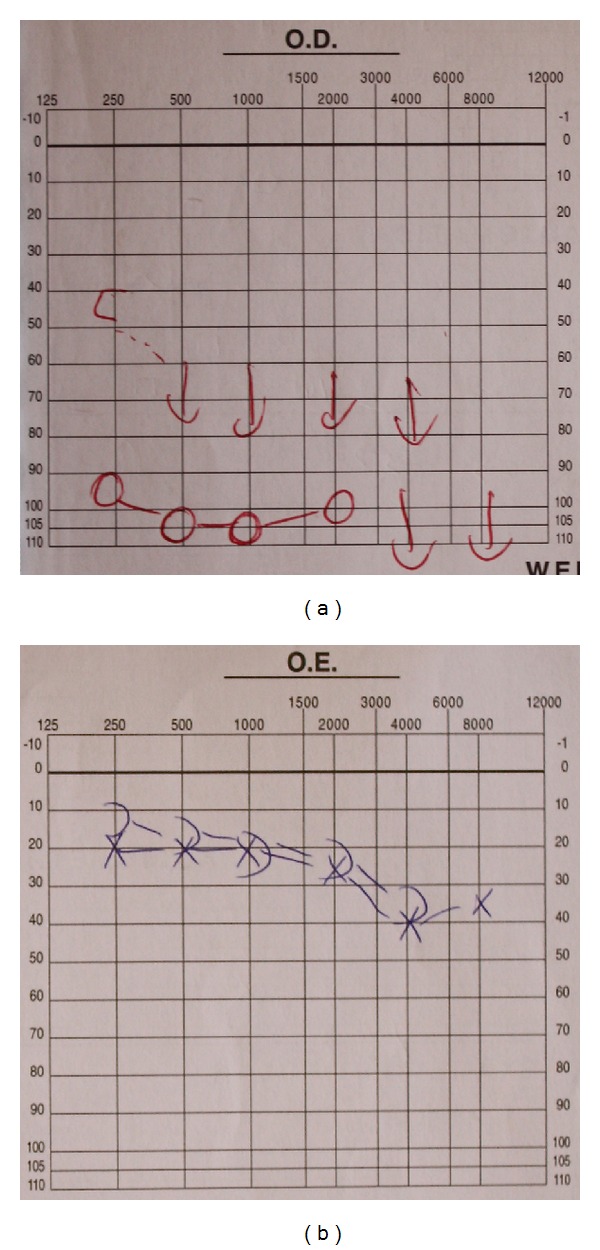
Audiogram showing complete hearing loss of right ear and no significant alterations of left ear.

The visual evoked potentials were within normal values. The audiogram showed a complete hearing loss on the right side and a normal exam on the left side (Figure 2).

A presumptive diagnosis of Rickettsiosis was considered. The patient was admitted to the hospital and treated with oral doxycycline (200 mg/day) and corticosteroids for 10 days, with improvement of the ocular symptoms and facial palsy. On discharge, the BCVA was 20/20 in both eyes, and there was residual hyperemia of the right optic disc.

Two weeks later, when the patient was on prednisolone 0,5 mg/kg/day, he complained of decreased visual acuity of the left eye. The BCVA was 20/20 in the right eye and 20/30 in the left eye. The ophthalmic examination only revealed mild edema of the left optic disc.

All the previous workup was repeated and addicionally a full-body PET scan was performed. The only positive test was the Weil-Felix reaction, but the IgM and IgG antibodies for *Rickettsia conorii* were negative.

It was decided to start a pulse of intravenous methylprednisolone (1 g/day for 3 days) followed by oral prednisolone 1 mg/kg/day in a tapering regimen. By the second day of intravenous steroids, the BCVA of the left eye improved to 20/20 and there was regression of the optic disc edema.

Excluding the infectious, neoplastic, granulomatous, and autoimmune etiologies and given the response of ocular manifestations to steroid treatment, we assumed this case as an atypical Cogan's syndrome.

**Figure 3:**
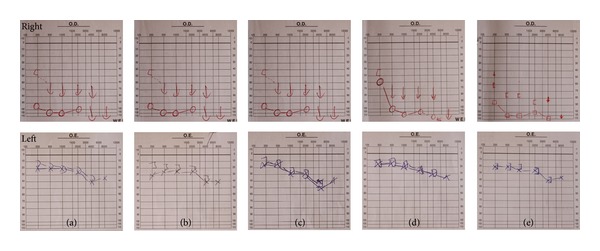
Evolution of right and left audiograms. There is complete right hearing loss from the beginning. Initially, the left ear audiogram was normal (a), (b), but the patient started to develop hearing loss at 4000 Hz frequency (audiogram (c)). (d) and (e) show recovering of left hearing after treatment.

Two weeks later, when the patient was on 0,75 mg/kg/day of prednisolone, he complained of decreased left hearing. The audiogram confirmed a loss of 5–10 db at 4000 Hz (Figure 3). It was decided to start immunosuppression with intravenous cyclophosphamide 1 g/month for 5 months and oral methotrexate 20 mg/week. There was improvement of symptoms after the second cycle with return of the audiogram to baseline values. 

Currently, 9 months after the last cycle of cyclophosphamide and on methotrexate 15 mg/week and prednisolone 5 mg/day, the patient is stable with no new visual or hearing complaints.

## 3. Discussion

Cogan's syndrome is a rare disorder. There are no specific tests for the diagnosis, which is based on the clinical presentation, medical history, and exclusion of other causes. Due to the variety of ocular and systemic inflammatory symptoms, there are numerous differential diagnoses (Table 1).

In this case, a positive Weil-Felix reaction was obtained, although with no rise of specific antibody titers for *Rickettsiae*. As all other diagnostic procedures were negative it was initially assumed as an infectious case. There are several reports of *Rickettsia conorii* infection with involvement of the central nervous system and the posterior segment of the eye [4–8]. Tsiachris and colleagues reported two cases of sensorineural hearing loss complicating severe rickettsial disease [9]. In a recent study [10], Kularatne and Gawarammana showed that the Weil-Felix test suffers from poor sensitivity and specificity (33% and 46%, resp.). As there was relapse of the symptoms along with the negativity of all other investigation, we assumed the case as an atypical CS. The positive Weil-Felix reaction was interpreted as a cross-reaction with other autoantibodies or other circulating autoimmune factors in the picture of an inflammatory state.

No treatment has proven very effective in CS. Corticosteroids often have good results on ocular, vascular, and visceral signs, but the effect on audiovestibular symptoms is not so effective, especially once deafness is present. Anterior ocular inflammation, such as IK, anterior uveitis, scleritis, and episcleritis, usually responds to topical corticosteroids [11]. Topical or systemic nonsteroidal anti-inflammatory drugs may be useful in patients with episcleritis and scleritis. Rarely, anterior inflammation may require systemic corticosteroid therapy [11, 12]. Posterior ocular involvement requires treatment with systemic corticosteroids, starting with a dose of prednisone of 1 mg/kg/day and slowly tapering. Failure to respond within 2-3 weeks of treatment, inability to taper the corticosteroid to a dose of 10 mg/day, or development of drug induced toxicity are indications for the use of other immunosuppressive drugs [11, 12].

In this case, as there was relapse of ocular and ear symptoms when reducing the dose of corticosteroids, there was the need to use immunosuppressive drugs. Currently there are no sufficient trials to recommend a specific drug, although good results have been obtained with methotrexate [3]. Our option was an approach with intravenous cyclophosphamide followed by oral methotrexate to enable tapering the corticosteroids. There was an improvement of all the symptoms except the right hearing loss.

When deafness is established it is almost always irreversible, although rare exceptions have been reported. Almost 90% of patients suffer severe hearing loss or total bilateral deafness, while ocular sequelae are rare [13]. The greatest long-term ophthalmologic risk in these patients is the development of cataracts from the administration of topical and/or systemic corticosteroids.

In addition to the ocular and audiovestibular symptoms, some patients may have aortitis or a large- or medium- to small-sized vessel vasculitis, which also require systemic immunosuppressive therapy. Aortitis may develop within weeks to years of disease onset and has been described in approximately 10%–12% of patients [14, 15]. It may cause proximal aorta dilation, aortic valve regurgitation, and thoracoabdominal aortic aneurysms. 

In summary, Cogan's Syndrome is a very rare clinical entity whose etiopathology is still unknown (infectious and immunological causes have been evoked). There are no specific biological tests for the diagnosis, and the investigations are aimed at excluding other etiologies. The treatment strategies are not clearly defined, and in some cases the atypical ocular manifestations make the diagnosis difficult delaying the treatment and resulting in profound bilateral deafness.

## Figures and Tables

**Table 1:** Differential diagnosis of Cogan's syndrome.

	Sarcoidosis
	Syphilis
	Vogt-Koyanagi-Harada syndrome
	Rheumatoid arthritis
	Whipple's disease
	Systemic lupus erythematosus
	Polyarteritis nodosa
	Wegener's granulomatosis
	Behçet disease
	Antiphospholipid antibody syndrome
	Ulcerative colitis
	Crohn's disease
